# Caligid sea lice (Copepoda: Caligidae) from golden snapper *Lutjanus johnii* (Bloch) in Australian waters, with the recognition of *Sinocaligus* Shen, 1957 as a junior synonym of *Caligus* Müller, 1785

**DOI:** 10.1007/s11230-023-10099-z

**Published:** 2023-06-21

**Authors:** Geoff Boxshall, Diane P. Barton

**Affiliations:** 1 Department of Life Sciences, Natural History Museum, Cromwell Road, London, SW7 5BD UK; 2 Department of Industry, Tourism and Trade, Fisheries Research, Berrimah, Darwin, NT 0828 Australia; 3 Natural Sciences Section, Museum and Art Gallery of the Northern Territory, Conacher Street, Fannie Bay, Darwin, NT 0801 Australia; 4 Present Address: Parasitology Section, South Australian Museum, North Terrace, Adelaide, SA 5000 Australia

## Abstract

Two species of sea lice are reported from the golden snapper *Lutjanus johnii* (Bloch) in Australian waters. One was represented by chalimus larvae, adult males and extremely slender females in which the genital complex is scarcely wider than the fourth pedigerous somite. These females are adult as they carry paired spermatophores and are identified as *Caligus dussumieri* Rangnekar, 1957 on the details of their appendages. *Caligus dussumieri* was formerly placed in the genus *Sinocaligus* Shen, 1957 but the characters supporting the validity of this genus are not robust, so it is here proposed to treat it as a junior subjective synonym of *Caligus* and transfer its species as: *Caligus formicoides* Redkar, Rangnekar & Murti, 1949, *Caligus dussumieri* Shen, 1957, *Caligus caudatus* (Gnanamuthu, 1950) **new combination** and *Caligus timorensis* (Izawa, 1995) **new combination**. All these species can be placed in the *C. bonito*-species group within *Caligus*. *Caligus rivulatus* Pilla, Vankara & Chikkam, 2012 is recognized as a junior subjective synonym of *C. dussumieri*. A new species, *C. auriolus* **n. sp.** is also described and this is placed in the *C. diaphanus* species-group. A key to species of this species-group is provided which indicates that *C. auriolus* **n. sp.** is most closely related to *C. stromatei* Krøyer, 1863 but the latter can be distinguished by the slender abdomen of the female and by the more complex myxal process on the maxilliped in the male.

## Introduction

The Caligidae Burmeister, 1835 is the most species rich family of parasitic copepods and, since caligid sea lice are one of the most important health hazards for farmed marine finfish (Johnson et al., 2004), it is of enormous commercial importance (Boxaspen et al., 2022). Boxshall (2018) reviewed historic data on caligids in Australian waters and found records of 69 species. He also reported another 16 species from Australia for the first time and described 13 new species from Moreton Bay, Queensland. The total number of caligid species known from Australian marine fishes after Boxshall’s (2018) contribution was 98. Since then, an additional species, *Lepeophtheirus spinifer* Kirtisinghe, 1937, has been reported from northern Australia (Diggles et al., 2021). Here we report on two further species, both taken from golden snapper, *Lutjanus johnii*, caught at various localities around the coast of the Northern Territory and Western Australia.

In addition to these caligids, copepods representing the families Lernanthropidae and Hatschekiidae were collected from the gills of the golden snapper. The two species of Lernanthropidae have been included in a separate larger study (Boxshall et al., 2020), while the hatschekiids have yet to be examined.

## Materials and Methods

Fish were collected by hook and line from several locations along the northern Australia coastline from Camden Sound in Western Australia to Townsville in northern Queensland. All fish were frozen prior to analysis: following defrosting, the gills and pharyngeal teeth plates were removed, separated and washed in water; gill arches and pharyngeal teeth plates were examined separately for the presence of parasites; the external surface of fish was not examined. The supernatant for the wash was removed and the detritus examined under a dissecting microscope. Individual fish were given DPIF (Department of Primary Industries and Fisheries) numbers.

The copepods were preserved in 70% ethyl alcohol. Prior to examination the specimens were cleared in lactic acid for at least 2 h and mounted on glass slides as temporary preparations. Limbs were dissected where necessary to observe fine details. Measurements were made using an ocular micrometer and drawings were made using a drawing tube on a Leitz Diaplan microscope equipped with differential interference contrast. Morphological terminology follows Boxshall (1990a) and Huys & Boxshall (1991); host fish names have been updated according to FishBase (Froese & Pauly, 2022).

The holotype of the new species is deposited in the collection of the Museum and Art Gallery of the Northern Territory (MAGNT) in Darwin; paratypes or voucher specimens of both species are deposited in the MAGNT and in the Natural History Museum, London.

## Systematics

*Caligus dussumieri* Rangnekar, 1957

Syn: *Sinocaligus dussumieri* (Rangnekar, 1957)

*Pseudopetalus dussumieri* (Rangnekar, 1957)

*Caligus rivulatus* Pilla, Vankara & Chikkam, 2012 **new synonym**

*Host*: *Lutjanus johnii* (Bloch)

*Material examined:* 6♀♀, 9♂♂ and 8 chalimus stages: 1♀, 1♂, 1 chalimus from DPIF 2297, Lorna Shoal, Northern Territory (12° 22.01′S 130° 17.37′E) on 05.12.2013 (MAGNT Reg.No. Cr019550); 1♂ from DPIF 2157, Darwin Harbour, Northern Territory (12° 39.02′S 130° 58.00′E) on 28.08.2013 (MAGNT Reg. No. Cr019551); 1♂ from DPIF 2268, Darwin Harbour, NT on 13.10.2013 (MAGNT Reg. No. Cr019552); 1♀ (damaged) from DPIF 1485, Melville Island, Northern Territory (11° 44.46′S 131° 16.89′E) on 23.08.2012 (MAGNT Reg. No. Cr019553); 1♀, 2♂♂, 2 chalimus from DPIF 2377, Nicoll Island, Northern Territory (13° 28.30′S 136° 16.64′E) on 10.12.2013 (MAGNT Reg. No. Cr019554); *Material in NHM, London*: 1 chalimus from DPIF 2590, Lorna Shoal, NT on 26.03.2014; 2 chalimus (dried) from DPIF 2270, Darwin Harbour, NT on 13.10.2013; 1♀, 1 chalimus from DPIF 2606, Elcho Island, Northern Territory (11° 55.13′S 135° 53.61′E) on 16.04.2014; 1♂ from DPIF 2372, Nicoll Island, NT on 02.12.2013; 1 chalimus from DPIF 2378, Groote Eylandt, NT on 13.10.2013; 2♂♂ from DPIF 2394, Camden Sound, Western Australia (16° 11.52′S 124° 32.52′E) on 11.09.2013; 1♀, 1♂ from DPIF 2391, Ord River, Camden Sound, WA on 11.09.2013; 1♀ from DPIF 2395, Ord River, Camden Sound, WA on 11.09.2013, NHMUK Reg. Nos. 2022.189-197.

**Fig. 1:**
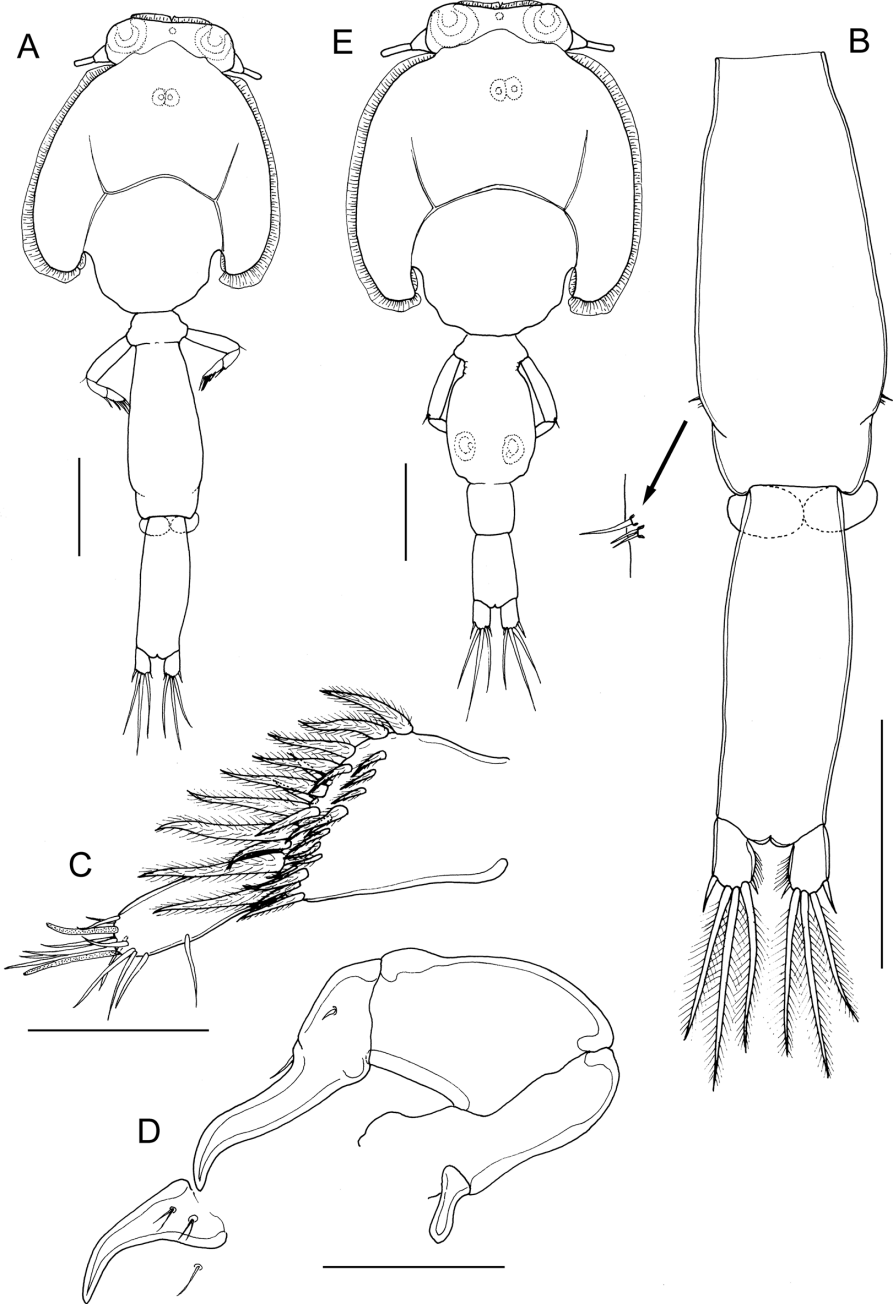
*Caligus dussumieri* Rangnekar, 1957. A. post-mating adult female bearing spermatophores, dorsal; B, genital complex and abdomen, dorsal; C, antennule; D, antenna and postantennary process *in situ*; E, adult male, habitus, dorsal. Scale bars: A, B, E, 500 µm, C, D, 100 µm.

*Description*: Adult female (Fig. 1A) mean body length 3.28 mm (range 3.05 to 3.65 mm), including caudal rami (based on 5 specimens). Cephalothorax about 1.2 times longer than wide; comprising about 46% of total body length. Free margin of thoracic portion of dorsal cephalothoracic shield extending posteriorly beyond rear margins of lateral portions. Large lunules present ventrally on frontal plates. Genital complex elongate, scarcely wider than fourth pedigerous somite, about 2.3 times longer than wide (0.87 x 0.38 mm); with near-parallel lateral margins; fifth legs located on lateral margins (Fig. 1B). Genital complex about 1.2 times longer than abdomen. Abdomen elongate, 1-segmented; about 2.6 times longer than wide (0.70 x 0.27 mm) (Fig. 1B); carrying paired caudal rami distally; anal slit terminal. Caudal rami with parallel sides, longer than wide. Each ramus armed with 3 long plumose setae on distal margin, short hirsute seta at inner distal angle, spiniform, hirsute seta sub-distally on outer margin, plus minute seta located just ventral to outer distal seta (not visible in Fig. 1B).

Antennule (Fig. 1C) 2-segmented; large proximal segment with 25 plumose setae arrayed along anteroventral surface and 2 setae located dorsally; distal segment bearing 12 elements (10 setae plus 2 aesthetascs) around apex, plus isolated seta on posterior margin. Antenna (Fig. 1D) comprising proximal segment bearing blunt-tipped, posteriorly-directed spinous process; middle segment subrectangular, unarmed; terminal segment forming weakly curved claw bearing short spine proximally, and minute seta on anterior margin. Postantennal process (Fig. 1D) weakly curved; ornamented with 2 bi-sensillate papillae on basal part and uni-sensillate papilla on adjacent ventral cephalothoracic surface.

**Fig. 2:**
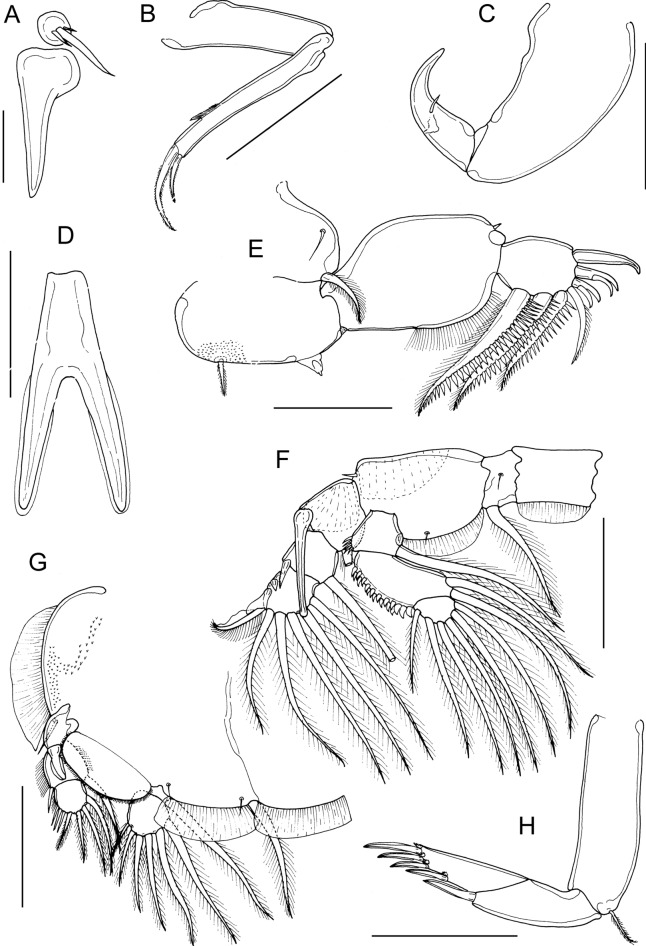
*Caligus dussumieri* Rangnekar, 1957, adult female. A, maxillule; B, maxilla; C, maxilliped, posterior; D, sternal furca; E, leg 1, F, leg 2; G, leg 3; H, leg 4. Scale bars: A, 50 µm, B, C, F-H, 200 µm, D, E, 100 µm.

Mandible of typical stylet-like structure, with 12 marginal teeth (not figured). Maxillule (Fig. 2A) comprising anterior papilla bearing 3 unequal, naked setae and posterior, tine-like process. Maxilla 2-segmented (Fig. 2B), comprising elongate, unarmed syncoxa and basis: basis bearing membranous subapical flabellum on anterior margin, and terminating in 2 subequal claw-like elements (calamus and canna). Calamus longer than canna, ornamented with strips of serrated membrane arranged obliquely around surface; canna ornamented with bilateral strips of serrated membrane. Maxilliped subchelate (Fig. 2C); proximal segment unarmed, with slight swellings on myxal surface; distal subchela with apical claw separated from proximal segmental part by incomplete suture; small seta present near concave margin.

Sternal furca (Fig. 2D) with divergent, pointed tines.

First swimming leg (Fig. 2E) with slender intercoxal sclerite; sympod with inner and outer plumose setae derived from basis; endopod represented by unarmed conical process on posterior margin of basis. Exopod short, robust, 2-segmented; directed laterally and forming main axis of leg; first segment broad, only about 1.5 times longer than wide and armed with small outer (anterior) spine and ornamented with row of setules along part of posterior margin; second segment short, 1.3 times longer than wide, armed with 3 long plumose setae along posterior margin, each ornamented with stout spinules laterally and slender pinnules medially; distal margin with 4 elements as follows: spine 1 (anterior-most) longest; spine 2 longer than spine 3, each with accessory process; seta 4 unilaterally plumose, longer than spine 1 and about as long as segment.

Second leg (Fig. 2F) biramous, with flattened protopodal segments and 3-segmented rami. Coxae of leg pair joined by intercoxal sclerite bearing marginal membrane posteriorly. Coxa with plumose seta posteriorly and surface sensilla. Basis armed with outer naked seta; ornamented with surface sensilla, marginal membrane posteriorly, and flap of membrane anteriorly, reflexed back over dorsal surface of segment. Exopodal segment 1 with long, straight, outer spine extending obliquely across ventral surface of segments 2 and 3, also armed with inner plumose seta and bearing flap of membrane anteriorly, reflexed back over dorsal surface of segment; segment 2 with short outer spine aligned parallel with longitudinal axis of ramus, and inner plumose seta; segment 3 with 2 outer spines, proximal spine small, distal spine with large flap of membrane; apical spine with marginal membrane laterally and pinnules medially, and 5 inner plumose setae. Endopodal segment 1 armed with inner plumose seta and ornamented with few slender spinules at outer distal angle; segment 2 ornamented with conspicuous denticles along outer margin, and bearing 2 inner plumose setae; segment 3 with 6 plumose setae.

Third leg pair forming flattened plate (apron) closing posterior part of cephalothoracic sucker as typical for genus. Leg (Fig. 2G) fused to plate-like intercoxal sclerite ornamented with marginal membrane posteriorly. Protopodal part flattened, bearing inner plumose seta at junction with intercoxal plate, and outer plumose seta located dorsally near base of exopod; single sensillae located adjacent to inner coxal seta and adjacent to origin of endopod; ornamented with strip of membrane along posterior margin medial to endopod and along lateral margin anterior to exopod. Exopod 3-segmented; first segment lacking inner seta, armed with weakly curved outer spine directed over ventral surface of ramus, spine ornamented with bilateral strips of membrane; second segment with small outer spine and inner plumose seta; third with 3 outer spines and 4 inner plumose setae; outer margins of segments 2 and 3 ornamented with slender setules. Endopod 2-segmented; first segment forming velum ornamented with row of fine setules along free margin and armed with inner plumose seta; compound distal segment with 6 setal elements increasing in length from outermost to innermost.

Fourth leg (Fig. 2H) 3-segmented, comprising slender protopodal segment and 2-segmented exopod: protopodal segment armed with plumose seta distally; first exopodal segment armed with slender outer spine; second with 1 lateral plus 3 distal spines; apical spine slightly longer than middle spine; middle and outer spines of similar length; each spine with pecten at base.

Fifth legs located posterolaterally on genital complex (Fig. 1B); each fifth leg comprising anterior process bearing short plumose seta (representing outer protopodal seta) and inner exopodal process armed with 2 plumose setae.

**Fig. 3:**
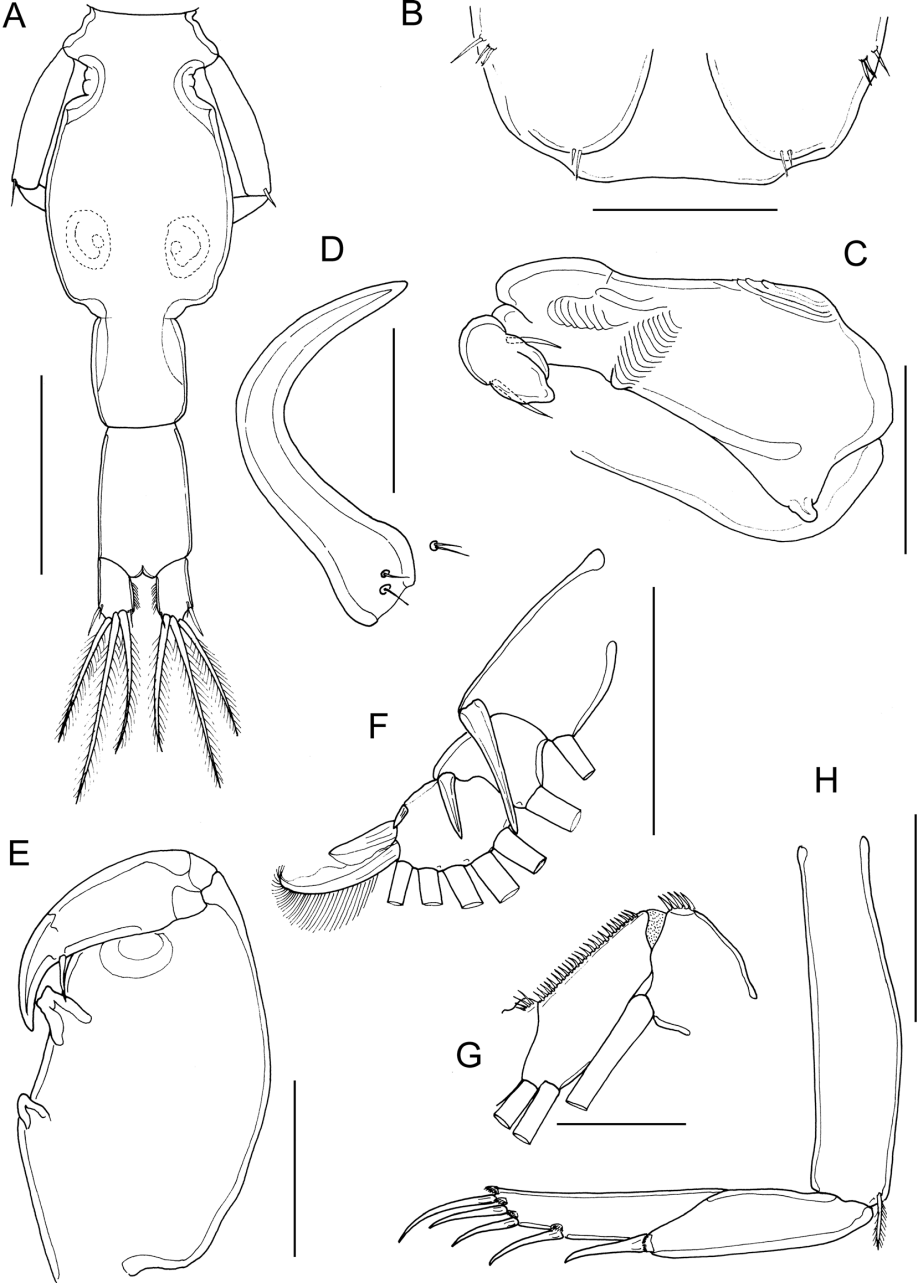
*Caligus dussumieri* Rangnekar, 1957, adult male. A, fourth pedigerous somite, genital complex and abdomen, dorsal; B, fifth legs and genital apertures, ventral; C, antenna; D, post-antennary process; E, maxilliped; F, exopod of leg 2; G, first and second endopodal segments of leg 2 showing ornamentation; H, leg 4. Scale bars: A, 500 µm, B, E, F, H, 200 µm, C, D, G, 100 µm.

Adult male (Fig. 1E) mean body length 3.02 mm (range 2.42 to 3.75 mm). including caudal rami (based on 10 specimens). Cephalothorax as in female. Genital complex (Fig. 3A) 1.3 times longer than wide (0.61 x 0.48 mm); with weakly convex lateral margins. Abdomen 2-segmented; first segment longer than wide (0.27 mm x 0.24 mm), second segment about 1.4 times longer than first, and about 1.6 times longer than wide (0.38 x 0.23 mm); carrying paired caudal rami posteriorly as in female.

Antennule, mandible, maxillule and maxilla as in female. Antenna modified (Fig. 3C); first segment elongate; second segment reflexed, elongate, bearing corrugated adhesion pads ventrally in distal part and anteriorly in proximal part; distal segment forming short flattened claw, armed with 2 setae proximally. Postantennal process (Fig. 3D) more strongly curved than in female; ornamented with sensillate papillae.

Maxilliped (Fig. 3E) with 2 processes on myxal margin of proximal segment; proximal process tooth-like, distal process larger, with truncate apex, opposing tip of subchela.

Leg 1 as in female. Leg 2 with outer spine on first exopodal segment less well developed than in female (Fig. 3F); spine on second segment directed obliquely across surface of ramus; endopod segment 2 with slender spinules along outer margin rather than robust denticles (Fig. 3G). Leg 3 as in female. Leg 4 (Fig. 3H) similar to female but apical spine slightly longer relative to middle and outer distal spines.

Leg 5 (Fig. 3B) represented by plumose, outer protopodal seta originating on papilla on somite surface and 2 plumose setae on inner papilla representing exopod. Sixth leg represented by plate closing off genital opening armed with 1 seta and 1 short spine on outer distal corner of genital operculum.

Remarks: The adult females collected from *Lutjanus johnii* had an extremely long and slender genital complex and abdomen (Fig. 1A, B). These females carried paired spermatophores and are identified as adults since female copepods become sexually receptive only after the final, definitive moult (Boxshall, 1990b). The extreme narrowness of the genital complex exhibited by these females is unique in *Caligus*, however, detailed examination of their appendages revealed a close resemblance to *Caligus biseriodentatus* Shen, 1957 and to *Sinocaligus dussumieri* (Rangnekar, 1957).

*Caligus biseriodentatus* was recognized as a member of the *Caligus bonito* species-group by Boxshall (2018), based on its possession of a 2-segmented exopod on leg 4 bearing 4 spines on the compound distal segment, combined with the presence of 3 plumose setae on the posterior margin of the distal exopodal segment of leg 1 in the female, plus the ornamentation of large denticles along the outer margin of the second endopodal segment of leg 2. The Australian material from *L. johnii* shares all of these features (as do all species currently placed in *Sinocaligus*), but can be distinguished from *C. biseriodentatus* by the length of the plumose setae on the posterior margin of the distal exopodal segment of leg 1. These setae are well developed and longer than seta 4 in the Australian material but are markedly shorter than seta 4 in female of *C. biseriodentatus* and are even further reduced in the male (Shen, 1957; Cressey & Cressey, 1980; Boxshall, 2018). In addition, the first exopodal segment of leg 4 is ornamented with conspicuous surface spinules in the female of *C. biseriodentatus* (see Cressey & Cressey, 1980; Pillai, 1985 (as *C. auxisi*); Boxshall, 2018) but the Australian material from *L. johnii* lacks such ornamentation.

*Sinocaligus dussumieri* was originally described, as *Caligus dussumieri*, based on a single ovigerous female found on the inside surface of the operculum of a clupeiform fish, *Dussumieria acuta* Valenciennes, 1847, caught off Mumbai (Rangnekar, 1957). Pillai (1968) redescribed the species, based on two females collected from the gills of *D. elopsoides* Bleeker, 1849 (as *D. hasseltii* Bleeker), and transferred it to the genus *Pseudopetalus* Pillai, 1962 as *P*. *dussumieri* (Rangnekar, 1957). However, as Boxshall & Montú (1997) noted, *Pseudopetalus* is a junior synonym of *Sinocaligus* Shen, 1957. In their major review of the family Caligidae, Dojiri & Ho (2013) accepted *S. dussumieri* as a valid species of *Sinocaligus*. The redescription of *S. dussumieri* by Pillai (1968) revealed several distinctive features of the swimming legs of this caligid: leg 1 has unusually short but broad exopodal segments and each of the plumose setae on the posterior margin of the distal segment is longer than seta 4 and is ornamented with spinules along its lateral margin; the first exopodal segment of leg 2 carries a distinctive, elongate outer spine with a spatulate tip; the proximal outer margin spine on the third exopodal segment of leg 2 is small and the distal spine on the same margin is ornamented with a large flap of membrane. These characteristics are all shared with our material from golden snapper and there is close agreement in almost all of the other appendages. The only exception is the female maxilliped, which Pillai (1985) showed as bearing a tapering process. No process was figured by Rangnekar (1957) and none was present in our Australian material. The process shown by Pillai (1985: Fig. 132E) is not in the normal position for a myxal process, i.e. opposing the tip of the claw, but it was also mentioned by Pillai (1968) and requires further investigation.

The above comparisons are focused on the detailed similarities in the appendages, however, there is an apparent major difference between the Australian material from *L. johnii* and the Indian material described by Rangnekar (1957) and Pillai (1968, 1985) from *Dussumieria* species, and that is the shape of the female body. The ovigerous females found by Rangnekar (1957) and Pillai (1968) all have an elongate and somewhat swollen genital complex and a laterally expanded abdomen. It is, largely, the laterally expanded abdomen that has been used as a generic level character to support the validity of the genus *Sinocaligus* (see Dojiri & Ho, 2013, for discussion). Variability in shape is apparent even between these ovigerous females: the female illustrated by Rangnekar (1957: Fig. 2a) has a genital complex with a slender anterior part (comprising 25% of the total length) and the abdomen is about 3.5 times longer than wide, whereas in Pillai’s (1968) females the slender anterior part is short and the abdomen is only about 1.8 times longer than wide (Pillai, 1968). The females from *L. johnii* are not ovigerous but they are adult and have mated as they carry paired spermatophores. It seems likely that the females of this caligid undergo a post-mating metamorphosis resulting in a major lateral expansion of both the genital complex and the abdomen. Such a post-mating metamorphosis in adult females is widespread in caligids (Boxshall & Özak, 2022). The extremely slender females figured here represent the immediate post-mating morphology while the laterally expanded ovigerous female figured by Pillai (1968) represents the fully metamorphosed adult. The adult figured by Rangnekar (1957) is also ovigerous but shows a lesser state of expansion.

Given the numerous detailed similarities between their appendages, we identify this material as conspecific with *Sinocaligus dussumieri* (originally described as *Caligus dussumieri*), and we infer that the differences in shape of the genital complex and abdomen are indicative of the state of development in the post-mating metamorphosis. Dojiri & Ho (2013) also noted variation in the shape of the female abdomen between typical *Sinocaligus formicoides formicoides* (Redkar, Rangnekar & Murti, 1949) and its variety *S. formicoides denticulatus* (Shen, 1957). Material from Hainan Island in the South China Sea described by Shen (1957) possessed a wide abdomen whereas the material from India had a slender spindle-shaped abdomen (Redkar, Rangnekar & Murti, 1949; Pillai, 1962). The females examined by Dojiri & Ho (2013) had an abdomen somewhat intermediate between these two states and they interpreted this variation as plasticity. We infer that this plasticity is largely a developmental phenomenon, with the lateral expansion of the abdomen becoming more pronounced in older adult females.

The discovery of the new material raises serious questions concerning the validity of the genus *Sinocaligus*. The main features distinguishing *Sinocaligus* from *Caligus* Müller, 1785 are the aliform lateral expansions on the abdomen (Dojiri & Ho, 2013), although their phylogenetic analysis also scored the presence of 7 caudal setae on the caudal ramus and the presence of 25 or 26 setae on the proximal segment of the antennule (Dojiri & Ho, 2013: Table XXIII). These last two features are doubtful. Their figure of the caudal ramus (Dojiri & Ho, 2013 Fig. 138d) showed only 5 setae but with 2 small cuticular markings which they interpreted as missing setae. Although the possession of 7 caudal setae is the ancestral state of the Copepoda (Huys & Boxshall, 1991), only 6 caudal setae is the maximum number found in any caligid. Since the caudal rami carry also sensory sensillae in some caligids (which also leave a similar marking in the cuticle when detached), we regard the evidence supporting the presence of 7 caudal setae in *Sinocaligus* as extremely weak. Similarly, the apparently reduced setal count on the first antennulary segment is not a robust character. The great majority of caligids carry 27 setae (25 anteroventral and 2 dorsal) on this segment but the more ventrally located setae can be small and densely packed, so observations can be difficult. The Australian material from *L. johnii* has the typical 6 caudal setae (one of them minute) and has 27 (25 + 2) setae on the first antennulary segment as found in the great majority of *Caligus* species. Both *Sinocaligus caudatus* (Gnanamuthu, 1950) and *Sinocaligus timorensis* (Izawa, 1995) also possess only 6 caudal setae (Gnanamuthu, 1950; Izawa, 1995). The number of setae on the first antennulary segment is 20 in the former and 26 in the latter, but neither description mentions any dorsal setae and both therefore seem unreliable.

The remaining character used to justify the generic status of *Sinocaligus* is the lateral expansion of the abdomen of the female, but our new evidence indicates that the expansion of the genital complex and of the abdomen is a late developmental phenomenon. The males exhibit no features that would differentiate them from a typical *Caligus* male. We, therefore, propose to treat the genus *Sinocaligus* as a junior subjective synonym of *Caligus* and transfer all of its species: *Sinocaligus formicoides* (Redkar, Rangnekar & Murti, 1949) returns to its original combination as *Caligus formicoides* Redkar, Rangnekar & Murti, 1949 and *Sinocaligus dussumieri* (Shen, 1957) returns to its original combination as *Caligus dussumieri* Shen, 1957. *Sinocaligus caudatus* (Gnanamuthu, 1950) becomes *Caligus caudatus* (Gnanamuthu, 1950) **new combination** and *Sinocaligus timorensis* (Izawa, 1995), originally established as *Pseudopetalus timorensis* Izawa, 1995, becomes *Caligus timorensis* (Izawa, 1995) **new combination**. All of these species can be accommodated within the *Caligus bonito* species-group to which *C. biseriodentatus* belongs. Interestingly, the original female of *C. biseriodentatus* illustrated by Shen (1957: fig. 114) has the same very slender genital complex and abdomen and is presumably at the same pre-metamorphic phase.

We also note here an additional new synonymy. Pilla et al. (2012) described a *Caligus* species collected from the body surface of *Lutjanus rivulatus* Cuvier, 1828 caught off the Visakhapatnam coast, India and considered it to be a new species for which they proposed the name *Caligus rivulatus*. Unfortunately, their publication was in an on-line only journal and neither the publication nor the proposed new name was registered with ZooBank or given an LSID number, and thus this is not a valid publication.

*Caligus rivulatus* is based on seven specimens which were all considered to be males by Pilla et al. (2012). However, the illustrated specimen is a female, as indicated by the slender subchelate form of the antenna, the lack of myxal processes on the maxilliped, and the unsegmented state of the abdomen. The slender form of the genital complex and abdomen indicate that this female is pre-metamorphic but it is adult as indicated by the presence of spermatophores which are shown with dotted lines on Pilla et al.’s dorsal habitus figure. Pilla et al.’s (2012) figures of the appendages of *C. rivulatus* show: leg 1 carries 3 plumose setae on the posterior margin of the distal exopodal segment, leg 4 is 3-segmented with a 2-segmented exopod bearing I, IV spines, and the outer margin of the second endopodal segment of leg 2 is ornamented with large denticles. This combination of character states is shared by members of the *Caligus bonito*-group.

*Caligus rivulatus* shares the same distinctive setation pattern for female leg 1 with *C. dussumieri* and *C. biseriodentatus*: spines 1 to 3 on the distal exopodal segment decrease in size from outer to inner, spines 2 and 3 each bear an accessory process, seta 4 is longer than spine 1 and longer than the segment, and the 3 plumose setae on the posterior margin are each ornamented with stout spinules laterally. However, *C. rivulatus* also possesses the same distinctive elongate outer margin spine with a spatulate tip, as found on the first exopodal segment of leg 2 in female *C. dussumieri*. In view of this and the numerous other similarities we make *C. rivulatus* Pilla, Vankara & Chikkam, 2012 available here and also recognize it as a junior subjective synonym of *Caligus dussumieri* Shen, 1957. The body length given for *C. rivulatus* by Pilla et al. (2012) was 2.36 to 3.12 mm, which overlaps with that of the pre-metamorphic material of *C. dussumieri* reported here (3.05 – 3.65 mm).

***Caligus auriolus*** **n. sp** ***.***

*Type Host*: *Lutjanus johnii* (Bloch)

*Type Locality*: Lorna Shoal, near Darwin, Northern Territory (12° 22.008′S 130° 17.366′E).

*Type Material*: Holotype female deposited in the collections of the MAGNT (Reg. No. Cr019547) plus 2♀♀ paratypes (Reg. No. Cr019548) and 5♂♂ paratypes (Reg. No. Cr019549); remaining 3♀♀, 9♂♂ and 13 damaged specimens and chalimus stages in NHM, London (NHMUK 2022.179-188).

*Material Examined*: Holotype ♀ from DPIF 2192, Lorna Shoal, NT (12° 22.01′S 130° 17.37′E) on 18.09.2013; 5 paratype ♂♂, 1 chalimus from DPIF 2573, Lorna Shoal, NT on 26.03.2014; 2 paratype ♀♀ from DPIF 2590, Lorna Shoal, NT on 20.03.2014; 2 paratype ♀♀, 2 paratype ♂♂, 2 chalimus from DPIF 2589, Lorna Shoal, NT on 10.03.2014; 2 paratype ♂♂ from DPIF 2590, Lorna Shoal, NT on 20.03.2014; 1 paratype ♂ from DPIF 2573, Lorna Shoal, NT on 26.03.2014; 1 chalimus from DPIF 1918, Lorna Shoal, NT on 28.06.2013; 1 paratype ♂, 3 chalimus from DPIF 2602, Elcho Island, Northern Territory (11° 55.13′S 135° 53.61′E) on 16.04.2014; 1 chalimus from DPIF 2620, Elcho Island, NT on 16.04.2014; 2 paratype ♀♀, 7 incomplete and chalimus stages from DPIF 2378, Nicoll Island, Northern Territory (13° 28.30′S 136° 16.64′E) on 10.12.2013; 1 paratype ♂ from DPIF 2377, Nicoll Island, NT on 10.12.2013; 1 paratype ♂ from DPIF 2374 Nicoll Island, NT on 02.12.2013.

*Etymology:* The specific name *auriolus* comes from the Latin, meaning golden, and refers to the common name of the host, the golden snapper.

**Fig. 4:**
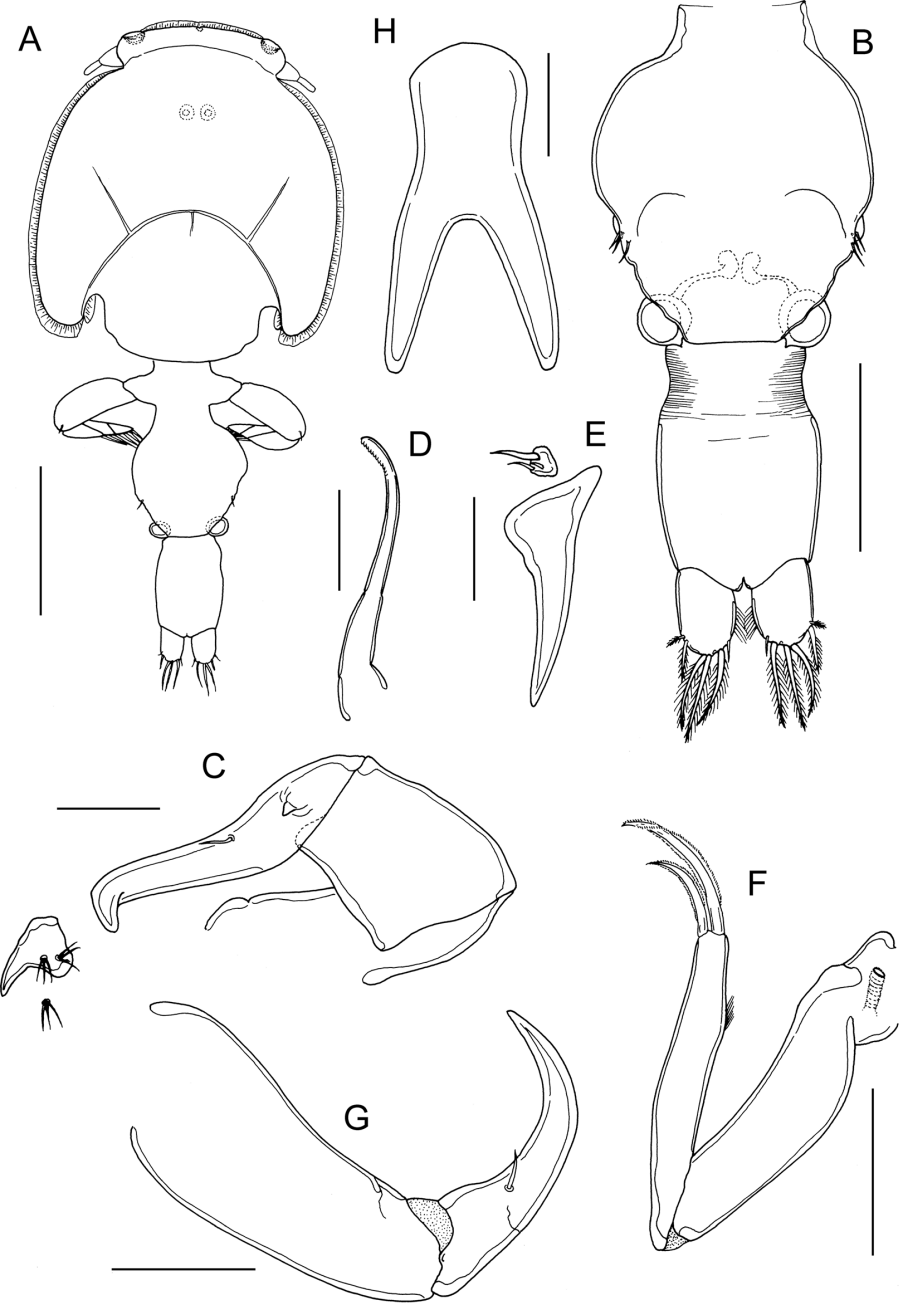
*Caligus auriolus* **n. sp.**, adult female. A, habitus, dorsal; B, genital complex and abdomen with spermatophores attached, dorsal; C, antenna and postantennary process *in situ*; D, mandible; E, maxillule; F, maxilla; G, maxilliped; H, sternal furca. Scale bars: A, 1.0 mm, B, 500 µm, C-E, H, 100 µm, F, G, 200 µm.

*Description*: Adult female (Fig. 4A) mean body length 4.18 mm (range 3.95 to 4.49 mm), including caudal rami (based on 5 specimens). Cephalothorax slightly longer than wide; comprising about 55% of total body length. Free margin of thoracic portion of dorsal cephalothoracic shield extending posteriorly beyond rear margins of lateral portions. Small lunules present ventrally on frontal plates. Genital complex about 1.2 times longer than wide (0.89 x 0.75 mm); with strongly convex lateral margins; fifth legs located on lateral margins (Fig. 4B). Genital complex about 1.35 times longer than abdomen. Abdomen elongate, 1-segmented; about 1.5 times longer than wide (0.66 x 0.45 mm) (Fig. 4B); anterior part of abdomen with transversely striated integument; carrying paired caudal rami distally; anal slit terminal. Caudal rami with parallel sides, longer than wide. Each ramus armed with 3 long plumose setae on distal margin, short hirsute seta at inner distal angle, spiniform, hirsute seta sub-distally on outer margin, plus small seta located just ventral to outer distal seta.

Antennule (not figured) typical for genus: comprising large proximal segment with 25 plumose setae arrayed along anteroventral surface and 2 setae located dorsally; distal segment bearing 12 elements (10 setae plus 2 aesthetascs) around apex, plus isolated seta on posterior margin. Antenna (Fig. 4C) comprising proximal segment lacking any posterior process; middle segment subrectangular, unarmed; terminal segment forming curved claw bearing short spine proximally and small seta on anterior margin. Postantennal process (Fig. 4C) reduced, with short tine and ornamented with 2 multi-sensillate papillae on basal part; similar multisensillate papilla present on adjacent ventral cephalothoracic surface.

Mandible (Fig. 4D) stylet-like, with 12 marginal teeth. Maxillule (Fig. 4E) comprising anterior papilla bearing 3 unequal, naked setae and tapering posterior process. Maxilla 2-segmented (Fig. 4F), comprising elongate unarmed syncoxa and basis: basis bearing membranous subapical flabellum on anterior margin, and terminating in 2 subequal claw-like elements (calamus and canna). Calamus longer than canna, ornamented with strips of serrated membrane arranged obliquely around surface; canna ornamented with strips of serrated membrane. Maxilliped subchelate (Fig. 4G); proximal segment with smooth myxal surface; distal subchela with apical claw separated from proximal segmental part by incomplete suture; small seta present near concave margin. Sternal furca (Fig. 4H) with divergent, pointed tines.

**Fig. 5:**
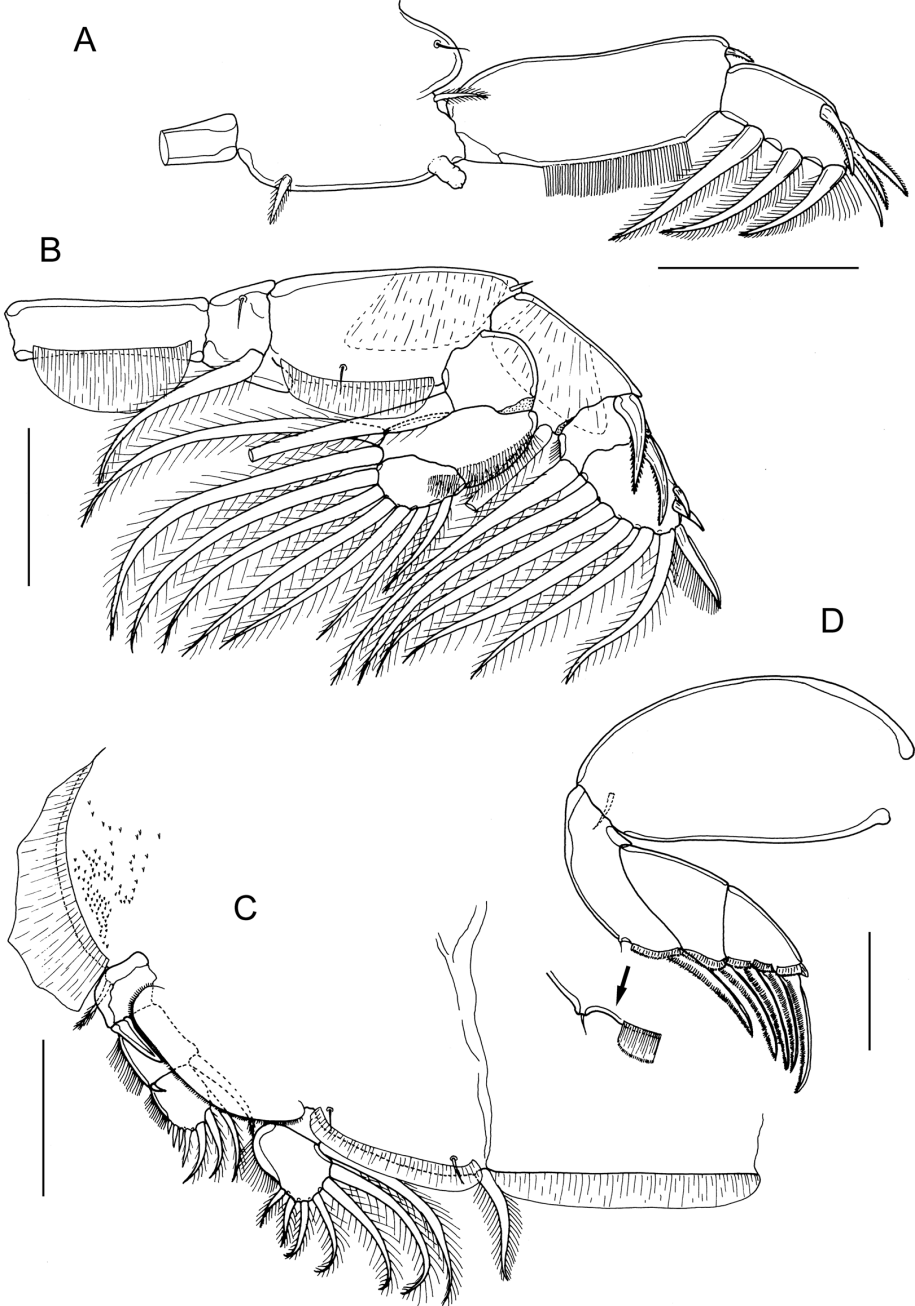
*Caligus auriolus* **n. sp.**, adult female. A, leg 1; B, leg 2; C, leg 3; D, leg 4 with inset showing marginal sensilla adjacent to end of linear pecten. All scale bars 200 µm.

First swimming leg pair (Fig. 5A) joined by slender intercoxal sclerite; sympod with inner and outer plumose setae derived from basis; endopod represented by unarmed process on posterior margin of basis. Exopod 2-segmented; directed laterally and forming main axis of leg; first segment about 2.5 times longer than wide and armed with small outer (anterior) spine and ornamented with row of setules along part of posterior margin; second segment about 2 times longer than wide, armed with 3 long plumose setae along posterior margin, and 4 distal elements along anterior and distal margins as follows: spines 1, 2 and 3 all of similar length, spines 2 and 3 lacking accessory process; seta 4 similar in length to spines 1 and shorter than segment.

Second leg (Fig. 5B) biramous, with flattened protopodal segments and 3-segmented rami. Coxae of leg pair joined by intercoxal sclerite bearing marginal membrane posteriorly. Coxa with plumose seta posteriorly and surface sensilla. Basis armed with outer naked spine, ornamented with surface sensilla and marginal membrane posteriorly, and with flap of membrane anteriorly, reflexed back over dorsal surface of segment. Exopodal segments 1 and 2 each with long, slightly-curved outer spine extending more-or-less parallel with main axis of ramus, each also armed with inner plumose seta; segment 1 also bearing flap of membrane anteriorly, reflexed back over dorsal surface of segment; segment 3 with 2 outer spines, proximal spine smaller than distal, both unornamented; apical spine with marginal membrane laterally and pinnules medially, and 5 inner plumose setae. Endopodal segment 1 armed with inner plumose seta, lacking ornamentation at outer distal angle; segments 2 and 3 both ornamented with patches of setules extending onto surface of segment; segment 2 armed with 2 inner plumose setae; segment 3 with 6 plumose setae.

Third leg pair (Fig. 5C) forming flattened plate closing posterior part of cephalothoracic sucker as typical for genus. Leg pair joined by plate-like, intercoxal sclerite (apron) ornamented with marginal membrane posteriorly. Protopodal part flattened, bearing inner plumose seta at junction with intercoxal plate, and outer plumose seta located dorsally near base of exopod; single sensillae located adjacent to inner coxal seta and adjacent to origin of endopod; ornamented with strips of membrane along posterior margin medial to endopod and along lateral margin anterior to exopod; Exopod 3-segmented; first segment armed with straight outer spine directed over ventral surface of ramus; second segment with small outer spine and inner plumose seta; third with 3 short outer spines and 4 inner plumose setae; outer margins of segments 2 and 3 ornamented with slender setules. Endopod 2-segmented; first segment forming long velum ornamented with fine setules along free margin and armed with inner plumose seta; compound distal segment with expanded outer margin and bearing 6 setal elements increasing in length from outermost to innermost.

Fourth leg (Fig. 5D) 4-segmented, comprising robust protopodal segment and 3-segmented exopod: protopodal segment armed with plumose seta distally; first exopodal segment armed with slender outer spine; second with 1 outer spine, third with 3 spines; all spines similar in length; each spine unilaterally fringed and with pecten at base expanded to form membranous strip along free margin of segment; first exopodal segment with minute sensilla on papilla on margin (Fig. 5D, insert) just proximal to strip-like pecten.

Fifth legs located laterally on genital complex (Fig. 4B); each fifth leg comprising anterior process bearing short plumose seta (representing outer protopodal seta) and inner exopodal process armed with 2 plumose setae.

**Fig. 6:**
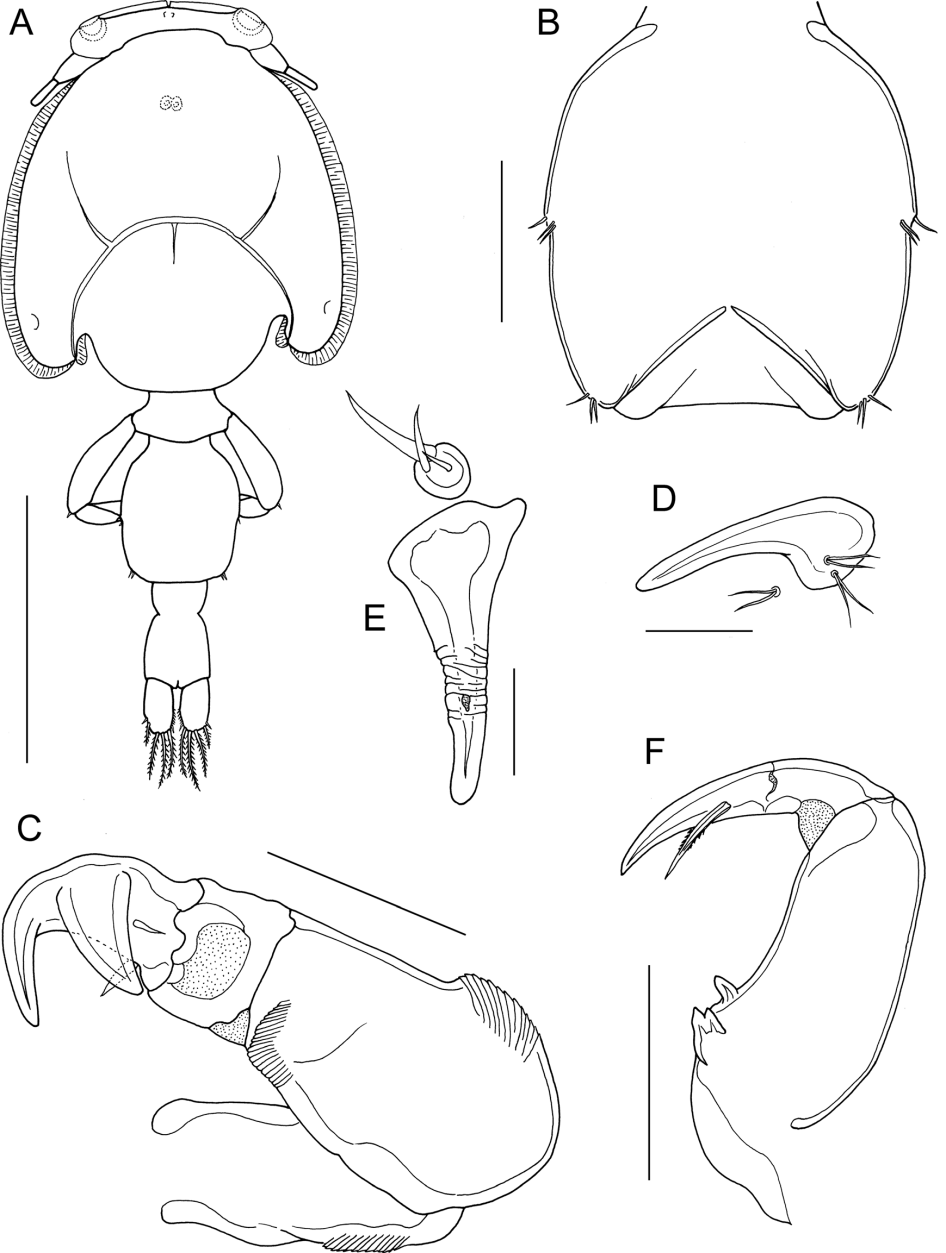
*Caligus auriolus* **n. sp.**, adult male. A, habitus, dorsal; B, genital complex, ventral view showing leg 5 and genital apertures; C, antenna; D, postantennal process; E, maxillule; F, maxilliped. Scale bars: A, 1.0 mm, B, 200 µm, C, 100 µm, D, E, 50 µm, F, 200 µm.

Adult male (Fig. 6A) mean body length 2.69 mm (range 2.50 to 2.93 mm) including caudal rami (based on 10 specimens). Cephalothorax as in female. Genital complex (Fig. 6B) about 1.1 times longer than wide (0.50 x 0.47 mm); with slightly convex lateral margins bearing fifth legs about at mid-level. Abdomen 2-segmented with segments separated by deep constriction; first segment wider than long (0.12 mm x 0.21 mm), second segment about 2.3 times longer than first, and about 1.1 times longer than wide (0.27 x 0.24 mm); carrying paired caudal rami distally as in female.

Antennule, mandible, and maxilla as in female. Antenna modified (Fig. 6C); first segment elongate; second segment reflexed, bearing corrugated adhesion pads ventrally in distal part and anteriorly in proximal part; distal segment with well-developed apical claw plus strong accessory process equal in size to claw, segment bearing 2 setae proximally. Postantennal process (Fig. 6D) with better developed tine than in female; ornamented with 2 bisensillate papillae and with similar bisensillate papilla on adjacent ventral cephalic surface. Maxillule (Fig. 6E) with surface corrugations in middle part of posterior process. Maxilliped (Fig. 6F) with 2 processes on myxal margin of proximal segment (syncoxa); proximal process with bifid apex, distal process tooth-like; seta on subchela longer than in female, extending almost to tip of claw.

Legs 1 to 4 as in female. Leg 5 (Fig. 6B) represented by outer protopodal seta originating on somite surface and 2 setae on inner papilla representing exopod. Sixth leg represented by opercular plate closing off genital opening armed with 3 slender setae on outer distal corner.

Remarks: The new species belongs to a group of species recognized by Boxshall (2018) as the *Caligus diaphanus*-group. Members of this group are characterised by the following combination of shared character states: 3-segmented exopod on leg 4 armed with I, I, III spines and typically ornamented with a linear strip of membrane (the modified pecten) associated with the base of each spine; three plumose setae present on posterior margin of distal exopodal segment of leg 1; spines 2 and 3 on distal exopodal segment of leg 1 lacking accessory processes; leg 2 with an ornamentation of fine setules extending from the margin over onto surface of both the second and third endopodal segments, and with the outer spines of the first and second exopodal segments aligned close to the longitudinal axis of the ramus; the female antenna lacking a posterior process on the proximal segment; and the tine on the post-antennal process vestigial or weakly developed. The males are typically characterised by the presence of a well developed accessory process on the distal claw of the antenna.

Boxshall (2018) established this group to include: *C. diaphanus* von Nordmann, 1832, *C. cybii* Bassett-Smith, 1898, *C. fajerae* Morales-Serna, Oceguera-Figueroa & Tang, 2017, *C. kanagurta* Pillai, 1961, *C. kapuhili* Lewis, 1967, *C. laticaudus* Shiino, 1960, *C. macrurus* Heller, 1865, *C. stromatei* Krøyer, 1863 (syn. *C*. *multispinosus* Shen, 1957), *C. pagelli* Delamare Deboutteville & Nuñes-Ruivo, 1958, *C. pelamydis* Krøyer, 1863*, C. platytarsis* Bassett-Smith, 1898, *C. robustus* Bassett-Smith, 1898, *C. rotundigenitalis* Yü, 1933, *C. tanago* Yamaguti, 1939, and *C. tenuis* (van Beneden, 1852). Boxshall & Bernot (submitted) noted that *C. pagri* Capart, 1941 also belongs in the *C. diaphanus*-group. Here we place the new species *C. auriolus* **n. sp.** in this group. These species can be distinguished with the aid of the following key to adult females:

1. Abdomen hyper-elongate, longer than cephalothorax and genital complex combined……2
  Abdomen shorter than cephalothorax and genital complex combined…………………………3
2. Sternal furca present; caudal rami 4.0 to 5.0 times longer than wide...……… *C. macrurus*
  Sternal furca absent; caudal rami 2.0 to 2.4 times longer than wide……………… *C. tenuis*
3. Maxilliped of female with large myxal process…………………………………….………….4
  Maxilliped of female with smooth myxal margin …………………………………………..…7
4. Abdomen less than half length of genital complex……………….………..……..*C. kapuhili*
  Abdomen more than 60 to 65% of length of genital complex ………….……………………5
5. Myxal region of maxilliped with large proximal process plus adjacent spiniform process on margin……………………………*C. robustus*
  Myxal region of maxilliped with large tapering process only on margin……………6
6. Abdomen longer than genital complex………………………………… *C. diaphanus*
  Abdomen shorter than genital complex………………………………………….*C. laticaudus*
7. Pectens on leg 4 exopod modified as hirsute digitiform processes….……….*C. kanagurta*
  Pectens modified as linear strips of membrane along margin of segments………..……...8
8. All exopodal spines on leg 4 about equal in length and directed away from ramus……....9
  Spine on first exopodal segment of leg 4 orientated in parallel with outer margin of second segment………………………………13
9. Outer margin spines on exopodal segments 1 and 2, plus proximal 2 spines on segment
  3 of leg 4 swollen and ornamented with short hairs over surface…..………10
  These spines on leg 4 slender and tapering towards tip, ornamented with strips of membrane and/or rows of setules……………………………11
10. Sternal furca with extremely flattened, spatulate tines that are wider than long; post-antennal process without tine…………………………*C. platytarsis*
  Sternal furca with tines longer than wide; post-antennal process with small tine…*C. tanago*
11. Abdomen less than 2 times longer than wide; protopodal segment of leg 4 swollen, about 2.0 times longer than wide…………………….*C. auriolus* **n. sp.**
  Abdomen about 3 times longer than wide; protopodal segment of leg 4 slender, about 2.5 times longer than wide………………………………12
12. Outer spine on first exopodal segment of leg 3 not reaching articulation between second and third segments; tine of vestigial post-antennal process shorter than base…………………………………………………………*C. pelamydis*
  Outer spine on first exopodal segment of leg 3 reaching beyond articulation between second and third segments; tine of vestigial post-antennal process longer than base………………………………………………….…..*C. stromatei*
13. Genital complex wider than long……………………………………………14
  Genital complex 1.25 times longer than wide……………………………*C. fajerae*
14. Abdomen markedly longer than genital complex, distinctly wider anteriorly and tapering evenly towards posterior ……………………*C. cybii*
  Abdomen shorter than or as long as genital complex…………………………………….15
15. First abdominal somite about 4 times longer than anal somite…………............*C. pagelli*
  First abdominal somite at most 2 times longer than anal somite……………………….16
16. Lateral margins of genital complex strongly rounded; outer spine on first exopodal segment of leg 3 reaching beyond articulation between second and third segments …………………………………C*. rotundigenitalis*
  Lateral margins of genital complex linear to slightly convex; outer spine on first exopodal segment of leg 3 not reaching articulation between second and third segments……………………………………………………………*C. pagri*

Within the *C. diaphanus*-group, the new species appears to be closely related to *C. pelamydis* and *C stromatei*. All three species share the possession of: a smooth myxal margin on the female maxilliped, pectens modified as linear strips on leg 4, and with all exopodal spines on leg 4 tapering to a pointed tip and directed outwards at an angle to the ramus. The female of *C. auriolus* **n. sp.** differs from *C. pelamydis* in its shorter abdomen, which is 1.5 times longer than wide compared to about 3.8 times longer in the latter species. Similarly, the male of *C. auriolus* **n. sp.** differs in having the anal somite about 1.1 times longer than wide compared to about 1.5 times in *C. pelamydis*. The form of the myxal process on the male maxilliped also differs between these two species. In male *C. pelamydis* the myxal process is a small, distally-bifid lobe (see Cressey & Cressey, 1980: fig. 66F) whereas in male *C. auriolus* **n. sp.** the myxal margin carries a simple distal process and a bifid proximal process.

Comparison of *C. auriolus* **n. sp.** with the description of *C. stromatei* in Ho & Lin (2004) (as *C. multispinosus*) reveals numerous close similarities even in fine details, such as the presence of a minute sensilla on the margin of the first exopodal segment of leg 4 in the female (Fig. 5D) just proximal to the linear, strip-like pecten and the unusually long seta on the male maxilliped (Fig. 6F). However, these species can be distinguished by: the proportions of the abdomen which is about 2.9 times longer than wide in *C. stromatei* but only 1.5 times longer in the new species; the shape of the sternal furca which has tapering divergent tines in the new species compared with rounded, more or less parallel tines in *C. stromatei*; and by the length of the spine on exopod segment 1 of leg 3, which extends beyond the articulation between segments 2 and 3 in *C. stromatei* but is short and does not reach this articulation in the new species. In the male of *C. stromatei* the maxilliped bears a tiny conical myxal process (Ho & Lin, 2004: Fig. 109E, as *C. multispinosus*) where in the male of *C. auriolus* **n. sp.** the myxal process comprises a simple distal process and a bifid proximal process. These differences are sufficient to differentiate between these two species.

## Discussion

Adult males and chalimus stages but only pre-metamorphic adult females of *C. dussumieri* were found on *L. johnii.* It is possible that the absence of metamorphosed females might simply reflect a change in microhabitat on the host but an alternative explanation is that a host switching event occurs during the life cycle, with the mated adult female switching to a *Dussumieria* species as a final host. Two host life cycles are uncommon in parasitic copepods, although species of some genera (but not all, see Ismail et al., 2013) within the family Pennellidae are confirmed as utilizing two hosts, including *Lernaeocera* de Blainville, 1822 which uses different fishes as both first and second hosts (Sproston, 1942), *Cardiodectes* Wilson, 1917, one species of which uses pelagic molluscs as the first host and fish as the second (Perkins, 1983), and *Pennella* Oken, 1815 which uses squid as first host and a marine vertebrate such as a whale or a fish, as second host (e.g. Pascual et al., 2001). However, life cycles involving two hosts have also been suggested for caligid copepods. Hayward et al. (2011), for example, found the developing chalimus stages of *Caligus chiastos* Ho & Lin, 2003 on *Thamnacornus degeni* (Regen) outside of sea cages used to farm southern bluefin tuna (*Thunnus maccoyii* (Castelnau)), while adults only were present on the farmed tuna within the cages.

Cressey & Cressey (1980) considered that species of *Scomberomorus* Lacépède, 1801 served as hosts only for immature stages of *Caligus biseriodentatus* and that adults appear to be found on a different host from the immature stages. They recognized the species originally described as *C. auxisi* by Pillai (1963) as the adult of *C. biseriodentatus* and the only host known to harbour an adult was *Auxis thazard* (Lacépède). Cressey & Cressey (1980) speculated that the adult may prefer a non-scombrid host but the recent discovery of two more adult females of *C. biseriodentatus* on *A. thazard* from Moreton Bay led Boxshall (2018) to infer that this scombrid may well be the preferred host of the adult.

It seems likely that *C. dussumieri* might be another example of a *Caligus* utilizing two hosts in its life cycle, with development in Australian waters taking place on *Lutjanus johnii* up to and including mating. After mating the fertilized adult female then switches to the second host, probably a clupeiform fish (given the known hosts of this species), where it completes its post-mating metamorphosis and commences egg string production. The new synonymy recognized here suggests that in Indian waters, *C. dussumieri* may use *Lutjanus rivulatus* as the first host before switching to a second host.
